# β2-agonists promote host defense against bacterial infection in primary human bronchial epithelial cells

**DOI:** 10.1186/1471-2466-10-30

**Published:** 2010-05-14

**Authors:** Claire A Gross, Russell P Bowler, Rebecca M Green, Andrew R Weinberger, Christina Schnell, Hong Wei Chu

**Affiliations:** 1 Department of Medicine, National Jewish Health, 1400 Jackson Street, Denver, Colorado, 80206, USA; 2 University of Colorado-Denver, 12800 E. 19th Avenue, Aurora, Colorado, 80045, USA

## Abstract

**Background:**

Airway epithelial cells are critical in host defense against bacteria including *Mycoplasma pneumoniae *(Mp) in chronic obstructive pulmonary disease (COPD) and asthma. β2-agonists are mainstay of COPD and asthma therapy, but whether β2-agonists directly affect airway epithelial host defense functions is unclear.

**Methods:**

Epithelial cells from bronchial brushings of normal (n = 8), asthma (n = 8) and COPD (n = 8) subjects were grown in air-liquid interface cultures, and treated with cigarette smoke extract (CSE) and/or Th2 cytokine IL-13, followed by Mp infection and treatment with β2-agonists albuterol and formoterol for up to seven days. Mp and host defense proteins short palate, lung, and nasal epithelial clone 1 (SPLUNC1) and β-defensin-2 were quantified. Expression of β2-adrenergic receptors was also measured by real-time quantitative RT-PCR.

**Results:**

(R)- or racemic albuterol and (R,R)- or racemic formoterol significantly decreased Mp levels in normal and asthma epithelial cells. Normal cells treated with Mp and (R)- or racemic albuterol showed an increase in SPLUNC1, but not in β-defensin-2. COPD cells did not respond to drug treatment with a significant decrease in Mp or an increase in SPLUNC1. IL-13 attenuated drug effects on Mp, and markedly decreased SPLUNC1 and β2-adrenergic receptors.

**Conclusions:**

These results for the first time show that β2-agonists enhance host defense functions of primary bronchial epithelial cells from normal and asthma subjects, which is attenuated by IL-13.

## Background

Bacterial infections are common in the airways of patients with chronic lung diseases [1-3]. As many as 40% of stable asthmatics test positive for atypical bacteria in airway samples[4]. Higher levels of pathogens such as *Mycoplasma pneumoniae *(Mp) and *Chlamydia pneumoniae *are associated with more severe asthma symptoms and increased COPD exacerbations[5,6]. Treatment with antibiotics such as clarithromycin can improve lung function in asthma patients with Mp[7]. However, bacterial infections remain prevalent in patients with chronic lung diseases, suggesting impaired lung host defense functions in these patients.

Innate immune response in airway epithelial cells provides a vital source of host defense molecules to protect against respiratory infection. For example, large airway epithelial cells constitutively produce short palate, lung, and nasal epithelium clone 1 (SPLUNC1), a member of the PLUNC protein family, which is proposed to exhibit host defense properties[8]. SPLUNC1 has been shown to possess antimicrobial and anti-inflammatory functions[9,10]. Bacterial infection increases SPLUNC1 levels. However, in an allergic setting including the Th2 cytokine IL-13, SPLUNC1 levels and Mp clearance are decreased[10]. Human β-defensins (hβDs) are another class of antimicrobial proteins produced by epithelial cells of airways and skin, and are able to kill a broad spectrum of bacteria including Mp[11]. hβD-3, closely related to hβD-2, is shown to be down-regulated by IL-13[12].

β2-agonists are the mainstay of therapy to induce bronchodilation in patients suffering from COPD and asthma. The drugs work through β2-adrenergic receptors to exert their functions including relaxation in smooth muscle cells[13]. The receptors are expressed in various types of cells in the lung including airway epithelial cells[14]. In addition to their bronchodilatory effect, β2-agonists have been shown to reduce the production of inflammatory cytokines and thereby airway inflammation[15]. Moreover, β2-agonist salmeterol was shown to protect airway epithelial integrity that was otherwise impaired by *Pseudomonas aeruginosa *exoproducts[16]. Interestingly, activation of β2-adrenergic receptors on human peripheral blood T cells could modulate production of Th2 cytokines (e.g., IL-13)[17]. In our previous studies, IL-13 was able to increase bacterial (e.g., mycoplasma) load in airway epithelial cells[10]. These publications suggest that β2-agonists may modulate airway epithelial cell host defense functions. However, there is a lack of direct evidence demonstrating host defense functions of β2-agonists in primary human airway epithelial cells.

Extended use of exogenous antibiotics may cause drug resistance, thereby becoming less effective at eliminating chronic infections that are prevalent in COPD and asthma patients. Therefore, it is beneficial to enhance airway production of endogenous antimicrobial substances to promote the clearance of invading bacteria. In this study, we seek to identify a novel function of the short- and long-acting β2-agonists albuterol and formoterol, in (R)-, (R,R)-, racemic, (S)-, or (S,S)-isomeric forms, which have different efficacies in bronchodilation [18-20]. Specifically, we hypothesize that β2-agonists possess an antimicrobial function by decreasing bacterial levels in primary human bronchial epithelial cells from normal subjects, asthmatics, and COPD patients. We predict that they do so in part through the induction of host defense molecules SPLUNC1 and hβD-2.

## Methods

### Study participants, bronchoscopy, and bronchial epithelial cell processing

Bronchoscopy with endobronchial epithelial brushings was performed on 24 human subjects (normal = 8, asthma = 8, COPD = 8). The clinical characteristics for all subjects are shown in Table 1. Four of the normal subjects were non-smokers and four were healthy smokers. Asthmatics met the American Thoracic Society (ATS) criteria for mild to moderate asthma. COPD patients had Global Initiative for COPD (GOLD) stages between II and IV. Bronchial brushings were performed as previously described[21] with a single-sheathed cytology brush (#CF-001, Medical Engineering Laboratory, Durham, NC). Up to six brushings were obtained per subject. Our research protocols were approved by the institutional review board at National Jewish Health, and all subjects provided written informed consent.

**Table 1:** Characteristics of human study subjects

	Age	Sex (M/F)	Smoking (pack-years)	FEV_1_, % predicted	FVC, % predicted	FEV_1_/FVC%
**Normal** (n = 8)	52.3 ± 4.3	4/4	**4 **- 0 **4 **- 37.0 ± 4.1	97.5 ± 6.8	92.0 ± 6.9	82.8 ± 1.3
**COPD** (n = 8)	66.8 ± 1.8	3/5	**8 **- 67.8 ± 10.8	38.5 ± 7.7	61.4 ± 7.9	46.6 ± 6.7
**Asthma** (n = 8)	48.8 ± 7.5	4/4	**5 **- 0 **3 **- 17.7 ± 6.7	82.1 ± 9.3	91.5 ± 8.0	69.5 ± 4.5

FEV_1_, forced expiratory volume in the 1^st ^second; FVC, forced vital capacity.

### Primary bronchial epithelial cell air-liquid interface cultures

**Figure 1:**
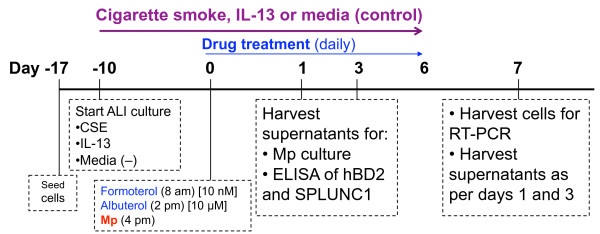
**Timeline of primary human bronchial epithelial cell air-liquid interface (ALI) cultures**. Cells were seeded in transwells in immersed culture for 7 days to allow cell proliferation, and then were shifted to ALI for 10 days to induce mucociliary differentiation. Cigarette smoke extract (CSE) and/or IL-13 treatment began with the ALI condition. At day 10, cells were treated with drug and infected with *Mycoplasma pneumoniae *(Mp). Apical supernatants were harvested at days 1, 3, and 7 post-infection, and cells were harvested at day 7 for mRNA analysis of gene of interest using real-time RT-PCR. SPLUNC1 = short palate, lung, and nasal epithelial clone 1; and hBD2 = human β-defensin-2.

Brushed bronchial epithelial cells were seeded onto 60 mm collagen-coated tissue culture dishes, and incubated at 37°C with 5% CO_2 _(Figure 1). At 80% confluence, they were transferred onto collagen-coated transwell inserts (4 × 10^4 ^cells/insert) in 12-well plates[21]. After reaching confluence under the submerged condition, they were shifted to air-liquid interface (ALI), and treated with or without IL-13 (10 ng/ml) and/or 20% cigarette smoke extract (CSE) to mimic the airways of asthmatics and smokers with or without COPD. IL-13 concentration was chosen based on previous publications in human airway epithelial cell cultures[10,21-23], and measurement of IL-13 protein in bronchoalveolar lavage fluid of asthmatics with a segmental allergen challenge[24]. Medium, IL-13, and/or CSE were replenished every 48 hours for 10 days to allow cell mucociliary differentiation. Cells were then infected with Mp and treated with (R)-, racemic, or (S)-albuterol (10 μM), or (R,R)-, racemic, or (S,S)-formoterol (10 nM) as shown in Table 2. β2-agonists were provided by the Sepracor, Inc (Marlborough, MA). The concentration of albuterol or formoterol was chosen based on previous publications in human airway epithelial cell cultures, and was consistent with the dose of effective therapy in humans [25-30]. On days 1, 3, and 7 post-Mp infection, an aliquot of apical supernatant was plated onto PPLO agar plates for Mp quantification. The remainder was stored at -80°C for quantification of SPLUNC1 and hβD-2 by ELISA. Seven days post-infection, cells were harvested into TRIzol (Invitrogen) for total RNA extraction.

**Table 2:** Air-liquid interface culture conditions (n = 56) of human primary brushed bronchial epithelial cells from normal subjects and patients with asthma and COPD

Drug	Medium	CSE	CSE + Mp	IL-13	IL-13 + CSE	IL-13 + CSE + Mp	IL-13 + Mp	Mp
No drug	x	x	x	x	x	x	x	x
(R)-albuterol	x	x	x	x	x	x	x	x
Racemic albuterol	x	x	x	x	x	x	x	x
(S)-albuterol	x	x	x	x	x	x	x	x
(R,R)-formoterol	x	x	x	x	x	x	x	x
Racemic formoterol	x	x	x	x	x	x	x	x
(S,S)-formoterol	x	x	x	x	x	x	x	x

CSE, cigarette smoke extract; Mp, *Mycoplasma pneumoniae*.

### Cigarette smoke extract (CSE) preparation

CSE was prepared daily as previously described with slight modifications[31]. Mainstream cigarette smoke was bubbled into 12.5 ml of serum-free cell culture medium. The medium was filter-sterilized, diluted in cell culture medium to 20%, and applied to the apical side of the transwells within 30 minutes of preparation.

### Mp preparation and culture

Mp was prepared for cell culture as previously described[10]. To quantify Mp, PPLO agar plates with epithelial apical supernatant were incubated at 37°C with 5% CO_2 _for seven days for colony forming unit (CFU) counting.

### Direct effects of β2-agonists on bacterial load in an epithelial cell-free environment

To determine if β2-agonists can directly affect bacterial growth, (R)-, racemic, (S)-albuterol (10 μM) or (R,R)-, racemic and (S,S)-formoterol (10 nM) or PBS (control) was incubated in quadruplicate with Mp (4 × 10^4 ^CFU per well, the same number of bacteria as plated on airway epithelial cells) in a cell-free 96-well plate. After 2 hours (a typical time for bactericidal assay), the supernatants were plated on PPLO agar plates to quantify the bacterial level.

### SPLUNC1 and hβD-2 ELISAs

Our previously developed direct SPLUNC1 ELISA was performed on the epithelial apical supernatants to quantify SPLUNC1[10].

hβD-2 levels in the apical supernatants were determined with an ELISA kit (PeproTech, Rocky Hill, NJ). The plate was coated with a capture antibody (Ab), blocked with BSA, and then incubated with apical supernatant, detection Ab and avidin-HRP. The plate was developed with 2,2'-Azino-bis(3-ethylbenzothiazoline-6-sulfonic acid).

### Real-time quantitative PCR

Reverse transcription and real-time PCR were used to determine the effect of IL-13 on mRNA expression of the β2-adrenergic receptor (ADRB2). 1 μg of total RNA was reverse transcribed with Oligo DT in a 50 μl reaction. A Taqman Gene Expression Assay (Applied Biosystems, Foster City, CA) was used to measure ADBR2 mRNA relative levels using the comparative threshold cycle (C_T_) method by normalizing to the housekeeping gene GAPDH (Applied Biosystems)[21].

### Statistical analysis

One-way analysis of variance (ANOVA) was used for multiple comparisons, and a Tukey's post hoc test was applied where appropriate. Student's *t *test was used when only two groups were compared. A p value ≤ 0.05 was considered significant.

## Results

### Impact of β2-agonists on airway epithelial bacterial load and SPLUNC1 levels in the absence of IL-13

All data presented is the average of the same conditions at days 1, 3, and 7 because the trends were consistent across all three-time points. We analyzed the data in smokers and non-smokers in each subject group, and did not observe any significant differences of Mp levels. Therefore, we combined the data from smokers and non-smokers for further analysis.

**Figure 2:**
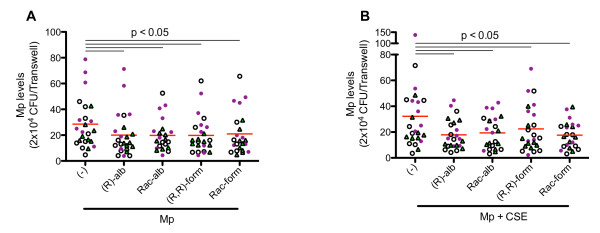
**Quantification of *Mycoplasma pneumoniae *(Mp) in the apical supernatants of cultured bronchial epithelial cells**. Cells from all three subject groups (n = 24) were treated with various isomers of albuterol and formoterol in the presence of Mp only (1 CFU/cell) (**A**), or Mp (1 CFU/cell) + CSE (20%) (**B**). Mp levels significantly decreased with by (R)-albuterol [(R)-alb], racemic albuterol [Rac-alb], (R,R)-formoterol [(R,R)-form], and racemic formoterol [Rac-form]. The red horizontal bars represent means of data under each cell culture condition. The open white circles indicate normal subjects; the closed purple circles indicate COPD patients; and the green triangles represent asthmatics.

To evaluate the overall cell responses to β2-agonists, we performed an initial data analysis in all 24 subjects from the three subject groups. (R)-albuterol, racemic albuterol, (R,R)-formoterol, and racemic formoterol significantly reduced Mp levels in the apical supernatants from cells treated with Mp (Figure 2A) or Mp + CSE (Figure 2B) as compared to cells not treated with any drug. (S)-albuterol and (S,S)-formoterol did not significantly alter Mp load (data not shown).

Differences in the drug effect on Mp load existed between subject groups. Among cells treated with Mp or Mp+CSE, normal subjects showed the strongest drug effect of decreased Mp burden, including significant (p < 0.05) reduction of Mp levels in four conditions ((R)-albuterol+Mp, (S)-albuterol+Mp, (R,R)-formoterol+Mp+CSE, and (S,S)-formoterol+Mp), as compared to non-drug-treated cells. Asthmatics showed a moderate trend, also with some significant (p < 0.05) decreases of Mp levels in three conditions ((R)-albuterol+Mp, (R,R)-formoterol+Mp, and Rac-formoterol+Mp). Cells from COPD patients exhibited the weakest trend for drug effect in decreasing Mp levels without statistical significance (Table 3).

**Table 3:** Effects of β2-agonist isomers on *Mycoplasma pneumoniae *(Mp) load in cultured human airway epithelial cells*

	Normal subjects		Asthma		COPD	
	**Mp**	**Mp + CSE†**	**Mp**	**Mp + CSE**	**Mp**	**Mp + CSE**

(R)-albuterol	**0.03**	0.16	**0.03**	0.24	0.43	0.14
Racemic albuterol	0.14	0.09	0.08	0.10	0.08	0.16
(S)-albuterol	**0.01**	0.48	0.15	0.21	0.44	0.20
(R,R)-formoterol	0.17	**0.03**	**0.04**	0.10	0.07	0.15
Racemic formoterol	0.48	0.10	**0.01**	0.37	0.13	0.08
(S,S)-formoterol	**0.01**	0.25	0.77	0.55	0.64	0.24

*, Data are presented as p values. A p value ≤ 0.05 is considered statistically significant. The p values were obtained by comparing β2-agonists with no drug treatments. †, CSE = cigarette smoke extract.

**Figure 3:**
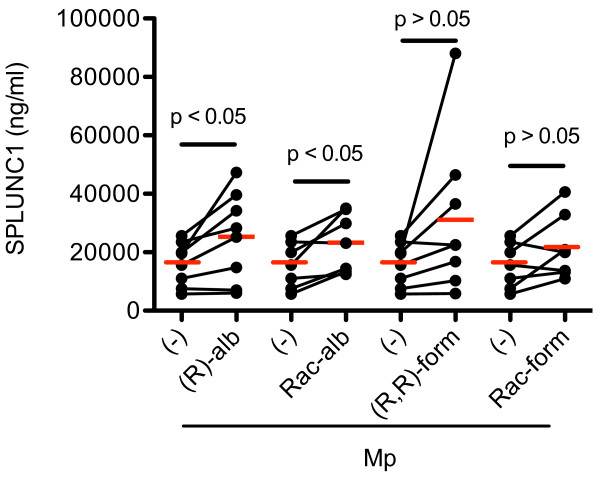
**SPLUNC1 protein levels in the apical supernatants of cultured bronchial epithelial cells**. Cells from normal subjects (n = 8) were treated with *Mycoplasma pneumoniae *(Mp, 1 CFU/cell). Both (R)-albuterol [(R)-alb] and racemic albuterol [Rac-alb] significantly increased SPLUNC1 levels. (R,R)-formoterol [(R,R)-form] and racemic formoterol [Rac-form] marginally increased SPLUNC1. The red horizontal bars represent means of data under each cell culture condition.

In all subjects combined, SPLUNC1 levels were not significantly different with the addition of any of the drugs. However, cells from normal subjects treated with Mp plus either (R)- or racemic albuterol significantly increased SPLUNC1 levels in the apical supernatant (Figure 3). (R,R)- and racemic formoterol marginally increased SPLUNC1 (p = 0.1 and p = 0.07, respectively). In addition, in Mp-infected normal subject cells, (S)-albuterol trended (p = 0.062) to increase SPLUNC1 protein levels. Cells from COPD and asthma patients did not show significantly increased SPLUNC1 following any drug treatment.

### Impact of β2-agonists on airway epithelial bacterial load and SPLUNC1 levels in the presence of IL-13

**Figure 4:**
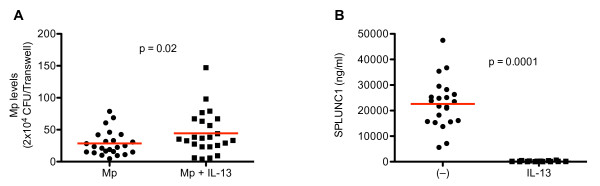
**Quantification of *Mycoplasma pneumoniae *(Mp) and SPLUNC1 protein levels in the apical supernatants of bronchial epithelial cells**. Cells from all three subject groups (n = 24) were treated with Mp in the presence or absence of IL-13 (10 ng/ml). IL-13 treatment significantly increased Mp levels (**A**), but dramatically decreased SPLUNC1 levels (**B**). The red horizontal bars represent means of data under each cell culture condition.

In sharp contrast to cells without IL-13 treatment, cells treated with IL-13 did not respond to any of the drugs with lower Mp levels. No clear trend of increase or decrease of Mp levels was present in the IL-13-treated cells with the addition of drug. Furthermore, overall Mp levels in IL-13 treated cells were significantly higher in all subjects combined (Figure 4A) or in any individual subject group (data not shown) compared to cells without IL-13. Accordingly, IL-13 treated cells had significantly lower SPLUNC1 levels than those without IL-13 (Figure 4B).

### Direct effects of β2-agonists on bacterial load in an epithelial cell-free environment

**Figure 5:**
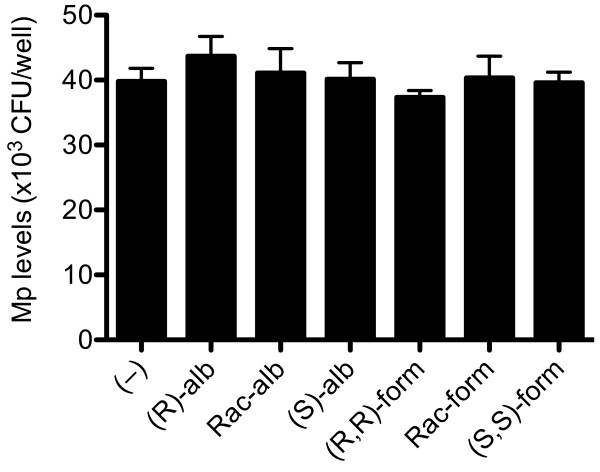
***Mycoplasma pneumoniae *(Mp) levels in the supernatants of a cell-free 96-well plate**. Various isomers of albuterol (10 μM) and formoterol (10 nM) or PBS (control, (-)) were incubated in quadruplicate with Mp (4 × 10^4 ^CFU per well) for 2 hours. Mp levels did not (p = 0.78) significantly change following treatments with (R)-albuterol [(R)-alb], racemic albuterol [Rac-alb], (S)-albuterol [(S)-alb], (R,R)-formoterol [(R,R)-form], racemic formoterol [Rac-form] or (S,S)-formoterol [(S,S)-form].

Albuterol or formoterol of any isomers, as compared to PBS (negative control), did not (p = 0.78) affect Mp levels in an epithelial cell-free environment (Figure 5). These data suggest that β2-agonists did not have a direct anti-bacterial (e.g., bactericidal) effect, and may exert their host defense activity through modulating airway epithelial cell functions.

### Impact of IL-13 on β2-adrenergic receptor (ADRB2) mRNA Levels

**Figure 6:**
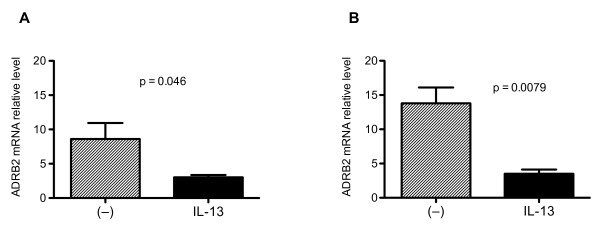
**Impact of IL-13 on β2-adrenergic receptor (ADRB2) mRNA expression in bronchial epithelial cells**. Compared with non-IL-13 treatment (-), IL-13 significantly lowered ADRB2 mRNA levels in cultured bronchial epithelial cells from asthma (**A**, n = 8,) and COPD (**B**, n = 8) patients.

Since cells treated with IL-13 failed to respond to β2-agonists, we determined if IL-13 reduced ADRB2 expression, rendering drug resistance. mRNA levels of ADRB2 were quantified in epithelial cells with or without IL-13 treatment. At baseline (no IL-13 treatment), bronchial epithelial cell ADRB2 mRNA levels were not (p = 0.15) significantly different among normal subjects (ADRB2 mRNA relative level: 15.0 ± 2.5), asthmatics (8.6 ± 2.3) and COPD patients (13.8 ± 2.3). Compared with the control (no IL-13), IL-13 treatment significantly decreased ADRB2 mRNA expression in bronchial epithelial cells from asthma (Figure 6A) and COPD patients (Figure 6B). However, IL-13 did not significantly (p = 0.12) reduce ADRB2 mRNA levels in normal cells.

### Impact of β2-agonists on hβD-2 levels

hβD-2 was measured to explain reduced Mp levels following β2-agonist treatment in epithelial cells. However, unlike the Mp or SPLUNC1 data, no significant differences were found in hβD-2 levels among any drug treatments, including samples with or without IL-13.

## Discussion

In the current study, we demonstrated a novel host defense function for the short- and long-acting β2-agonists albuterol and formoterol in primary brushed human bronchial epithelial cells. We found that the drugs were capable of lowering the Mp burden (up to 40% reduction) of epithelial cells, and that they appeared to do so in part through the induction of SPLUNC1, especially in normal subjects. However, in the COPD cells and in all the cells treated with IL-13, this drug effect was abolished, possibly through the reduction of SPLUNC1 and β2-adrenergic receptor (ADRB2).

### Host defense function of β2-agonists in the absence of IL-13

A certain population of asthmatics has no or very low Th2 cytokine background[32,33]. Therefore, testing the effects of β2-agonists without IL-13 is relevant for this subset of patients. (R)- and racemic albuterol and (R,R)- and racemic formoterol were found to reduce Mp levels in human bronchial epithelial cells mainly from normal subjects and/or asthmatics without IL-13 treatment. Our results suggest that β2-agonists enhance host defense functions in the airway mucosa. This decrease was significant with all 24 subjects combined in both Mp-only and Mp+CSE conditions. When data were analyzed in individual groups of subjects, the cells from the three subject groups responded differently to drug treatment following Mp infection. A previous study shows that mucin MUC5AC mRNA and protein levels can be differentially regulated in normal versus asthmatic brushed bronchial epithelial cells *ex vivo*[34]. The current study finds COPD cells responded less effectively to β2-agonists compared with normal and asthmatic cells. In response to β2-agonists, the normal cells had the lowest bacterial load; asthmatic cells also had significantly lower Mp burden. However, the COPD cells showed only a slight trend of decrease in Mp levels. Overall, the COPD cells carried a higher Mp burden than normals or asthmatics, suggesting that the COPD cells had inherently suppressed host defense against Mp as compared to the other groups. For example, in the current study, we found that baseline SPLUNC1 levels in apical supernatants of airway epithelial cells from COPD patients (18073 ± 2577 ng/ml) trended to be lower than those from normal subjects (24631 ± 2676 ng/ml, p = 0.14) and asthmatics (25020 ± 4480 ng/ml, p = 0.22).

While the racemic isomers of β2-agonists are used clinically, the (R)- and (R,R)- isomers are known to be the drugs' active forms. Application of (S)-albuterol and (S,S)-formoterol resulted in reduced Mp load only in normal cells, but not cells from asthmatics and COPD patients. (S)-albuterol has traditionally been thought to be inert, but studies have shown that the (S)-isomer may actually increase inflammation and negate the anti-inflammatory effects of (R)-albuterol[18,19]. It is therefore not surprising to observe differing antimicrobial effects of (S)- and (S,S)-isomers in cells from various study groups (e.g., normal subjects versus asthma and COPD patients). The mechanisms by which (S)- or (S,S)-isomers differ from (R)- or (R,R)-isomers in reducing bacterial levels remain unclear, and will be addressed in our future studies. For example, a β2-receptor antagonist can be used to test whether different isomers act similarly through β2-receptors.

How β2-agonists increase airway epithelial host defense functions remains unclear. Our data demonstrate that albuterol resulted in an increase in secreted SPLUNC1 levels in normal cells treated with Mp alone. This corresponded to the decrease in Mp burden seen in these cells. However, no significant increase of SPLUNC1 existed in the cells treated with both Mp and CSE. Further, there was only a slight induction of SPLUNC1 following drug treatment in the asthma cells and none in the COPD cells. These data indicate that the heightened host defense can be explained in part, but not entirely, through increased SPLUNC1 levels, particularly in normal cells. Conversely, no significant differences were detected in hβD-2 levels in any of the conditions, suggesting that hβD-2 may not contribute to increased host defense through β2-agonists. Other mechanisms may also contribute to the beneficial effects of β2-agonists on airway epithelial defense against bacterial infection. For example, Hasani and colleagues demonstrated that albuterol treatment in COPD patients enhanced lung mucociliary clearance[35], but whether this is linked to less bacterial load in the lung was not investigated. Moreover, long-acting β2-agonist salmeterol was shown to attenuate *Pseudomonas aeruginosa*-induced mucosal damage in cultured human nasal turbinate tissue[36]. Future studies are warranted to: (1) further explore the mechanisms by which albuterol or formoterol exerts host defense functions. These include, but are not limited to, studies aimed at dissecting the involvement of β2-adrenergic receptor vs. other receptors such as platelet-activating factor (PAF) receptor and β1-adrenergic receptor[37]; and (2) determine if β2-agonists are able to reduce lung bacterial load in COPD patients, especially those with acute exacerbations.

We are aware that our results in human cells do not support those of a mouse study conducted by Maris and colleagues[38], who found worsened clearance of nontypeable *Haemophilus influenzae *with salmeterol treatment in mice. Several differences exist between the two studies, including the types of cells cultured *in vitro *(primary human bronchial epithelial cells versus mouse alveolar macrophage cell line MH-S), the bacteria studied (atypical bacteria versus gram negative bacteria), and the drugs used. Therefore, a more systematic human study is needed to further investigate the in vivo effects of β2-agonists on airway mucosal innate immunity. We also realize that the concentration of albuterol in our study may be high. Future studies are needed to accurately measure the concentration of albuterol in airway epithelial lining fluid of human subjects who receive albuterol, especially the more active R-isomer.

### Host defense function of β2-agonists in the presence of IL-13

Because a Th2 cytokine milieu exists in both asthma and COPD, it is important to determine if β2-agonists in the presence of a Th2 cytokine such as IL-13 function as efficiently as in the absence of a Th2 cytokine. IL-13 was shown to dampen epithelial cells' host defense response, as overall Mp levels were higher in the IL-13-treated cells than those without IL-13. To explore the underlying mechanisms for the loss of effect of β2-agonists in IL-13-treated cells, we measured SPLUNC1 levels. We found that SPLUNC1 levels in the IL-13 treated cells were markedly lower than those in non-IL-13 treated cells, suggesting that the presence of IL-13 suppressed cells' ability to clear bacteria. None of the drugs lowered Mp levels in this condition. Therefore, IL-13 attenuated the effects of β2-agonists on lowering Mp levels. To further investigate the mechanism by which IL-13 inhibited host defense, we quantified the mRNA levels of ADRB2 from samples cultured with and without IL-13. Treatment with IL-13 significantly reduced ADRB2. This may provide an additional explanation why the IL-13-treated cells did not show any significant drug effect. Our results, for the first time, provide evidence that a Th2 cytokine IL-13 can significantly decrease ADRB2 expression. This may have clinical implications to improve the efficacy of β2-agonist therapy. For example, a therapy such as corticosteroid treatment, aimed at reducing the Th2 cytokine production or activity will be required to improve the antimicrobial efficacy of β2-agonists in both asthma and COPD patients.

## Conclusions

The goal of current study was to examine if β2-agonists, the mainstay of therapy in COPD and asthma, exert host defense functions in airway mucosa. We found that in normal bronchial epithelial cells without exposure to a Th2 cytokine IL-13, β2-agonists albuterol and formoterol decrease Mp load. The antimicrobial effect of albuterol may be in part through the induction of SPLUNC1 from airway epithelial cells, but not through its direct bactericidal activity on Mp. This effect is attenuated in COPD cells and particularly by IL-13 treatment. Future studies are needed to determine whether the use of a corticosteroid in addition to the β2-agonist can rescue the host defense functions of airway epithelial cells in COPD cells and in cells with IL-13 treatment. The combination of a long-acting β2-agonist with a corticosteroid may be most beneficial for enhancing endogenous host defense functions of airway epithelial cells in COPD and asthma patients with a Th2 cytokine background. Additionally, it may be possible to increase the ADRB2 levels in patients to enhance the effectiveness of β2-agonists. Long-acting β2-agonists such as formoterol do not have the same desensitizing properties as short-acting agonists[13]. This concept is further supported in our current study in that ADRB2 expression was significantly decreased by approximately 2-fold in (R)-albuterol-treated bronchial epithelial cells compared to negative controls (p = 0.045), but there was no difference between (R,R)-formoterol-treated cells and negative controls. Although further mechanistic studies are needed to reveal the interplay among short-acting β2-agonists such as (R)-albuterol, ADRB2, Mp load and SPLUNC1, our current study suggests that the use of a long-acting β2-agonist may not only induce bronchodilation, but also increase airway epithelial innate immunity in patients with a lung disease such as asthma.

## Competing interests

The authors declare that they have no competing interests.

## Authors' contributions

CAG, ARW and RMG performed airway epithelial cell cultures, ELISA and PCR. RBP did the bronchoscopy and bronchial brushings. HWC is the principal investigator of the research projects, and develop the research ideas and experimental design. CAG, RBP and HWC were involved in manuscript writing and editing. All authors read and approved the final manuscript.

## Pre-publication history

The pre-publication history for this paper can be accessed here:

http://www.biomedcentral.com/1471-2466/10/30/prepub
